# Use of a Continuous Glucose Monitor to Determine the Glycaemic Index of Rice-Based Mixed Meals, Their Effect on a 24 h Glucose Profile and Its Influence on Overweight and Obese Young Adults’ Meal Preferences

**DOI:** 10.3390/foods11070983

**Published:** 2022-03-28

**Authors:** Khadidja Chekima, Benjamin Tziak Ze Wong, Mohd Ismail Noor, Yasmin Beng Houi Ooi, See Wan Yan, Brahim Chekima

**Affiliations:** 1 Faculty of Health and Medical Sciences, Taylor’s University, Subang Jaya 47500, Selangor, Malaysia; khadijachekima@gmail.com (K.C.); yanseewan@gmail.com (S.W.Y.); 2 Faculty of Social Sciences & Leisure Management, Taylor’s University, Subang Jaya 47500, Selangor, Malaysia; tziakze.wong@taylors.edu.my; 3 Faculty of Medicine and Health Sciences, The National University of Malaysia, Bangi 43600, Selangor, Malaysia; ismailnoor49@gmail.com; 4 Faculty of Food Science and Nutrition, University Malaysia Sabah, Kota Kinabalu 88450, Sabah, Malaysia; yasmin@ums.edu.my; 5 Faculty of Business, Economics and Accountancy, University Malaysia Sabah, Kota Kinabalu 88450, Sabah, Malaysia

**Keywords:** continuous glucose monitoring (CGM), glycaemic index, overweight and obesity, young adults, postprandial glucose response, rice variety

## Abstract

Postprandial hyperglycaemia is associated with an increased risk of type-2 diabetes. This study aims to determine the glycaemic index (GI) of three varieties of rice-based mixed meals and their effects on glycaemic variability (GV), 24 h mean glucose levels and target ranges, and rice variety preferences among overweight and obese young adults using real-time continuous glucose monitoring (rtCGM). In a randomised controlled crossover design, 14 participants (22.8 ± 4.6 years, 32.9 ± 5.8 kg/m^2^) were randomly assigned to receive 3 rice-based mixed meals containing 50 g of available carbohydrates (white rice meal = WRM; brown rice meal = BRM; and parboiled basmati rice meal = PBRM) and 50 g of a glucose reference drink on alternate days. GI, GV, 24 h mean glucose levels and target ranges were measured. Rice variety preferences were compared with those of baseline data and determined at the end of the study period. Results: The analysis found that PBRM was low in GI (45.35 ± 2.06), BRM medium in GI (56.44 ± 2.34), and WRM high in GI (83.03 ± 2.19). PBRM had a significantly (*p* < 0.05) lower 24 h mean glucose level, higher in-target 24 h glucose level percentage and non-significantly (*p* > 0.05) lower GV compared to WRM. Prior to observing their postprandial glucose levels generated by rtCGM, the participants preferred WRM (64.3%) over other meals, whereas this preference changed significantly (*p* < 0.05) at the endpoint (PBRM, 71.4%). PBRM reduced 24 h glucose level and GV of overweight and obese young adults. The rtCGM is proven to be reliable in measuring GI, while providing robust continuous glycaemic information. This may serve as an educational tool that motivates eating behaviour changes among overweight and obese young adults.

## 1. Introduction

The latest statistic pertaining to overweight and obesity among adults aged 18 and above is 1.9 billion, equivalent to 39% of the world’s total population [1]. A total of 90% of those with type 2 diabetes are obese or overweight [2]. The World Health Organisation (WHO) declared obesity as a disease in 2000 and has reached epidemic proportions worldwide in 2020 [3]. Due to the seriousness of this condition in recent years, more countries around the world are acknowledging this declaration and altering their approach to tackle this disease [4,5,6]. 

Obesity is mainly caused by modifiable lifestyle practices, such as excessive energy intake and physical inactivity [3]. Excessive energy intake has been partially attributed to the consumption of a large percentage of high glycaemic index (GI) and glycaemic load (GL) carbohydrates [7]. Controlling GI by substituting high GI with lower GI foods may improve satiety, thereby reducing calorie consumption and lowering glycaemic response, insulinemia, and the risk of type-2 diabetes. 

Rice is the second highest consumed staple food in the world, supplying 16.5% of the global calorie intake [8] and providing up to 66% of the daily energy intake in Southeast Asia, where the prevalence of obesity has recently increased [9]. The GI of rice varies greatly from low-to-high GI, depending on the different varieties [10]. Rice is usually consumed as part of a meal that may contain vegetables, poultry, seafood, and meat, which are the sources of proteins and fats that may influence the GI of rice [11,12]. GI is usually measured in individual foods rather than in meals. Other confounding factors that could influence the GI of food, such as the presence of proteins and fats, are not well accounted for when this technique is applied and thus may not reflect the actual way this staple food is consumed. The method of estimating meals using a formula instead of directly measuring meals’ GI can overestimate the meals’ GI by between 22% and 50% [13]. Additionally, it is suggested that the measured GI cannot be generalised to all populations as studies in the literature proved that there can be significant variations in the glycaemic responses and GI of foods according to ethnicity [14,15]. Their findings challenge the assumption that a single GI value is adequate to characterise the glycaemic potential of a food across all population groups or is independent of the consumer. The above findings also suggested the need to evaluate the GI of foods as they are consumed in meals among specific populations. 

GI is usually measured using the standard methods, which have several drawbacks. With the robustness of the continuous glucose monitoring (CGM) system, it can be utilised to measure the glycaemic response to foods and be able to overcome the shortcomings of the other common classical methods [16,17]. The individualisation and real-time data of 24 h glucose levels generated by rtCGM allow a more extensive assessment of daily glucose variation [18] as compared to the classical methods. 

The aim of the present study is to determine the GI of three varieties of rice-based mixed meals commonly consumed in Malaysia (white rice, brown rice and parboiled basmati rice) using rtCGM, and their effect on the 24 h glucose profile of overweight and obese young adults. It also aims to determine the influence of the participants’ knowledge of postprandial glucose data generated by rtGCM has on their preference for rice varieties among this population group, which may indicate the potential use of rtCGM as an educational tool in selecting lower GI foods as part of a dietary intervention to manage obesity and prevent diabetes. 

## 2. Materials and Methods

### 2.1. Study Design

This was a randomised, controlled crossover non-blinded study that was conducted at Taylor’s University, Malaysia. The study was approved by Taylor’s University Human Ethics Committee (TUHEC) (HEC 2019/071) and was conducted in accordance with the guidelines of the Helsinki Declaration. It was registered with the UMIN Clinical Trials Registry as UMIN000045385. 

Individuals who provided their consent underwent the screening process, which was performed based on inclusion and exclusion criteria. The inclusion criteria were overweight or obese individuals (BMI ≥ 25 kg/m^2^), 18 to 35 years old, non-smoking, non-alcoholic, stable body weight (±3 kg) for the past 3 months, and normal fasting blood glucose (<5.6 mmol/L). The exclusion criteria were pregnant or lactating women, following a vigorous weight loss regimen, diabetic, suffering from hepatic or renal dysfunction, suffering from any gastrointestinal diseases, having had bariatric surgery, taking supplementation or medication that affects appetite, food intake, or glucose metabolism (e.g., corticoids, thyroid hormones, and thiazide diuretics) [19].

During screening, individuals were asked about their preference between three types of rice-based meals namely, white rice meal (WRM), brown rice meal (BRM), and parboiled basmati rice meal (PBRM). Upon recruitment, the anthropometric and body composition of the participants were measured. The participants were required to wear light clothing and all measurements were conducted in triplicate. Standing height without shoes was measured by using Seca 213 Stadiometer to the nearest 0.1 cm. Body composition was measured using the Tanita DC-360 Body Composition Analyser. Once the participants’ characteristics were obtained, the participants had the rtCGM sensors placed on their upper arm. Participants consumed a reference drink (glucolin) and three mixed meals of a low, medium, and high GI, randomly on alternate days to allow for a one day washout period between each meal. The reference drink was consumed twice, whereas the mixed meals were each consumed once. Randomisation of the meals’ consumption was generated by an online software: random.org/sequences/, accessed on 18 January 2021. During the study period, participants were advised to not increase their physical activities and to not vary the type of food or meals consumed before starting a 10 h overnight fasting period prior to the test days. After the arrival to the study centre between 8 am to 9 am of the test days, they were allowed to rest for 10 min before they began the testing of the food. They were instructed to consume the mixed meals within 15 min and were allowed to drink 250 mL of plain water. The postprandial glucose levels were automatically recorded every 15 min via the rtCGM sensor worn by the participants. A nutritionist monitored the participants ensuring all test foods were consumed accordingly. They were then allowed to carry on with their normal daily routine.

Once the consumption of all the mixed meals and reference drink is completed, the glucose data that were displayed in the form of a line graph indicating the postprandial glucose levels were explained to the participants. The participants were able to observe the differences in postprandial glucose levels in response to the consumption of the different meals consisting of different varieties of rice. They were educated on low GI and GL diets and how rtCGM can provide real-time information of their glucose levels in response to their dietary carbohydrate intake. They were then asked once again about their preference between the three meals and were required to provide reasons for their selection. A follow up survey was conducted three months after the trial to assess the longer-term commitment of the participants. Participants were asked about their practice of rice selection for their daily consumption. 

### 2.2. Mixed-Meals and Reference Drink

Three rice-based mixed meals were served with grilled chicken (100 g) and salad (100 g). The nutritional information and the serving sizes of the mixed meals were tabulated, as shown in Table 1. All mixed meals were served in isoglycaemic proportions of 50 g of available carbohydrate from rice [11]. The three meals consisted of three different types of rice, namely white rice, brown rice, and parboiled basmati rice, which are reflective of high, medium, and low GI rice varieties, as is stated in www.glycemicindex.com, accessed on 16 December 2020 by the University of Sydney. Participants were required to consume 50 g of glucose anhydrous powder dissolved in 250 mL of water as a control [20]. The nutritional compositions of the rice varieties were obtained from their respective manufacturers, whereas the nutritional composition of chicken and salad were obtained from Nutritionist ProTM software. The test meals were prepared by Deux Foods where all the meals’ specific descriptions, methods of preparation and portion sizes were provided to the named caterer. The meals were freshly prepared on the test days, as it was reported that the cooling of white rice for 24 h reduced its glycaemic response [21].

### 2.3. Continuous Glucose Measurement 

The rtCGM system used in this study is FreeStyle Libre by Abbott Diabetes Care, Alameda, CA, U.S.A. FreeStyle Libre provides real-time continuous glucose data automatically measured every 15 min, for 14 days via an rtCGM sensor. The sensor contains glucose oxidase impregnated in the sensor electrode, which translates the interstitial glucose into a signal via a chemical reaction. Information regarding the glucose levels is then transmitted in real-time, wirelessly to an rtCGM transmitter where the information can be observed and downloaded for analysis. FreeStyle Libre sensor is readily calibrated during the manufacturing process. The sensors were inserted in the upper arms of the participants [22]. Participants wore the sensors for an average of 12 days and were required to scan the sensor at least every 8 hours to transmit the glucose data to the reader for storage.

The common metric used to determine the accuracy of CGM systems is the mean absolute relative difference (MARD). The lower the MARD percentage, the more accurate the system is compared to plasma glucose reference. The FreeStyle Libre system has been shown to have a MARD of 12.8%, when compared to the plasma glucose reference [23]. The different metric used to demonstrate the clinical relevance of a glucose monitoring system is known as the Error Grid Analysis (EGA). There are two types of error grids that are frequently used: the Consensus Error Grid, also known as the Parkes Error Grid, and the Clarke Error Grid. Both Error Grids indicate whether CGM system findings are clinically acceptable, depending on their proximity to a reference glucose value. The Error Grids are divided into zones ranging from A to E. Clinically acceptable results are those in zones A and B, whilst outcomes outside of these zones may have an unfavourable clinical effect. The greater the proportion of findings falling between zones A and B, the more clinically accurate the glucose system. For the Freestyle Libre system, 99.7% of glucose values fall within Zone A or Zone B of the Consensus Error Grid [24].

### 2.4. Statistical Analyses

Previous studies reported that a total of 11 participants are sufficient to detect a 15% change in area under the 24 h glucose curve with 85% power significant at 0.05 [25,26], where gender did not affect the 24 h glucose outcomes [27]. All statistical analyses were conducted using Statistical Package for the Social Sciences (SPSS) software Version 27.0. Before conducting any further analysis, the normality of data was tested using the Shapiro–Wilk test. Data are presented as the means and standard deviation, unless stated otherwise. Statistical results were considered significant at *p* < 0.05. Postprandial glucose levels of 24 h of each meal were extracted from the CGM reader to calculate the GI of meals using the standarised equation (GI of test food = Incremental area under the curve (IAUC) of test food/ IAUC of reference food × 100). For a comparison between the glucose measurement of mixed meals at different time points, repeated measure analysis of variance (ANOVA) with Tukey’s post hoc test were performed. To determine the mean difference between the mixed meals’ effects on 24 h daily glucose level, 24 h glucose target ranges and glycaemic variability (% CV), analysis of variance (ANOVA) with Tukey’s post hoc test were conducted.

## 3. Results

### 3.1. Participants Characteristics

**Figure 1:**
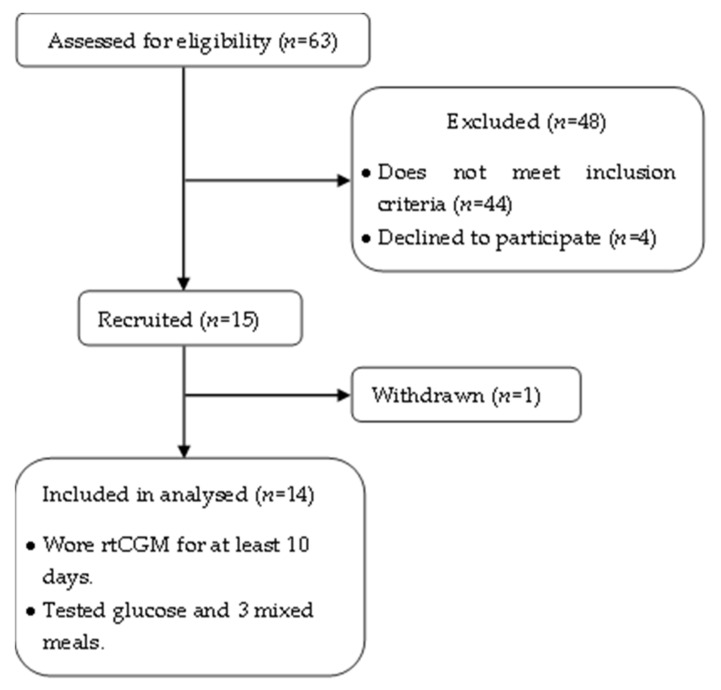
Consort flow chart.

Based on the screening assessment, those who fulfilled the inclusion criteria and gave their consent were recruited. Out of the 15 participants enrolled in the study, one participant withdrew herself from the study due to her inability to commit her time to the study (Figure 1). The characteristics of the participants are shown in Table 2. 

### 3.2. Glycaemic Index of Mixed Meals

The postprandial glucose levels of the reference drink and mixed meals were measured at 15 min intervals over a duration of 2 hours using an rtCGM. The IAUC of the glucose response curves indicate a significant difference (*p* < 0.05) between all three meals (Table 3). The GI of the mixed meals was calculated based on the IAUC of the obtained interstitial glucose values. By having a glucose solution at a reference value of 100, the estimated GI values of WRM, BRM and PBRM were 83.03 ± 2.19, 56.44 ± 2.34 and 45.35 ± 2.06, respectively. The measured meals’ GIs of the three rice varieties are relatively close to their respective predicted meal’s GI. The measured GI and predicted GI classified WRM as high GI, BRM as medium GI and PBRM as low GI. 

### 3.3. Mixed Meals’ Effects on 24 h Glucose Profile

To observe whether there is an effect of consuming different varieties of rice with different GI values on the overall glucose level within a day, a 24 h mean glucose level after the consumption of the mixed meals was measured using an rtCGM. The glucose level was highest for the reference drink (5.37 ± 0.54 mmol/L), followed by WRM (5.29 ± 0.62 mmol/L), BRM (5.03 ± 0.45 mmol/L), and the lowest was PBRM (4.87 ± 0.66 mmol/L) (Table 4). Reference drink, WRM, and BRM had a relatively similar 24 h mean glucose level as there was no significant difference between these values (*p* > 0.05). On the contrary, PBRM, which has the lowest GI value, showed a significantly lower (*p* < 0.05) mean glucose level, compared to the reference drink and WRM and was not significantly different from BRM. 

The 24 h glucose levels of the reference drink and mixed meals were then analysed further by determining the percentage in different target levels, as shown in Table 4. WRM had the lowest percentage in the target range with a total percentage of 85.6 ± 17.3%. This in-target percentage was relatively similar to that of the reference drink (86.9 ± 14.1%) and BRM (88.5 ± 12.2%), as it did not indicate a significant difference based on the *p*-value (*p* > 0.05). This value however was significantly lower (*p* < 0.05) than that of PBRM (90.1 ± 9.6%). 

Glycaemic variability (GV) of the reference drink and mixed meals was calculated as the coefficient of variation (CV%) of the mean readings over 24 h. The CV has the benefit of being a metric related to the mean, which makes it more expressive of hypoglycaemic excursion in comparison to SD alone. A higher value of GV indicates a greater fluctuation of blood glucose levels. Based on the analysis, reference drink has the highest value of 20.8 ± 5.2% and PBRM has the lowest value of 17.2 ± 4.7%, although the differences are not significant (*p* > 0.05). 

### 3.4. Rice-Based Mixed Meal Preference

Preceding the consumption of the mixed meals, participants were asked which mixed meal they preferred consuming. As indicated in Table 5, more than half (64.3%) of the participants prefer WRM. These participants then consumed the three mixed meals and observed their personalised postprandial glucose levels continuously in real-time using rtCGM in response to the mixed meals consumed. Post consumption, they were once again asked about their preference of the mixed meals. At this point, the percentage of distribution changed drastically. No participant prefers consuming WRM, whereas 71.4% prefer PBRM (Table 5). These percentage distributions indicated a significant difference pre and post consumption at *p* < 0.05. Based on an open-ended question, most of the participants stated that the underlying reason for the change of preference was due to the observed postprandial glucose effect indicated by the rtCGM. This preference was supported by the dietary intake observed among the participants. After observing the real-time postprandial glycaemic responses, the frequency of the postprandial glucose level above 7.8 mmol/L reduced by an average of 61.5 ± 20.3% among participants. During follow-up, out of 12 participants that responded, 33.3% reported selecting lower GI rice (either brown rice or basmati rice) for their daily consumption. A total of 41.7% of the participants reported switching back and forth between lower and higher GI rice varieties, and the remainder 25% reported that it was difficult to make changes to the variety of rice consumed. 

## 4. Discussion

The postprandial glucose levels of the meals were measured using rtCGM and the IAUC was calculated for all the meals. In general, WRM showed a significantly greater increase in the postprandial glucose level compared to BRM and PBRM. Despite the consumption of the white rice with the protein and fat sources from the side dishes, the glycaemic response of the white rice was not greatly reduced and was classified as a high GI meal. Generally, the three varieties of rice had similar GI classifications when measure as single foods and when measured as a mixed meal containing chicken and vegetables, as our results are in line with previously published GI categories of the named rice varieties [10,28,30]. When comparing the measured GI of the meals using rtCGM, the values were closely similar to the predicted GI values of the meals. 

Using the rtCGM system, we were able to identify meals of different GIs accordingly in a group of overweight and obese young adults. This indicates that the approach of using rtCGM in measuring GI of foods is shown to be reliable in this study, as the results are comparable to those published in the literature, which were measured using standardised methods [10,28,30] and to those which were calculated by using the formula. In contrast to the classical methods of measuring GI over a 2 h period, CGM may provide crucial information relevant for unravelling the role of GI in an individual’s glucose profile that is continuously measured over 24 h. The glucose level does not drop back to the baseline after a 2 h period for all foods [31], highlighting the importance of evaluating these levels beyond the 2 h period, which is conducted in the standardised method. It has been proven that CGM system had no significant difference in postprandial glycaemic responses, when compared to venous and capillary blood glucose levels [17]. Relative to the classical methods used currently, rtCGM is more comfortable and convenient for both the tested individuals and researchers as it is simpler and less invasive, making it more preferable and acceptable.

Among the three meals, only PBRM significantly alleviated the 24 h glucose level of the overweight and obese young adults. This indicates that the inclusion of a lower GI meal in the daily diet of this group of individuals has the potential of attenuating their daily mean glucose level, in line with normal weight respondents [20]. According to the analysis of the 24 h glucose target range, PBRM had the highest percentage of in-target range and the lowest percentage below and above the target range. This portrays that lower GI food may increase the time it takes the glucose level to be in the normal range, which is translated as having better glucose control. 

The insignificantly lower GV induced by the low GI meal in this study is contrary to that obtained in a study among normal weight participants [32,33], which reported a significantly lower GV when low GI foods were consumed. In our study, only a single low GI meal was consumed in a day in comparison to four low GI meals (breakfast, snack, lunch, and dinner) consumed within a day in the previous studies. The great difference in the low GI food consumption pattern between these studies may have contributed to the difference in the outcome. Consuming four meals a day consisting of low GI foods may not be common practice. Nevertheless, all studies indicated the potential of lower GV in response to consuming lower GI foods. Thus, practicing a lower GI diet may produce a more prevalent difference in GV as compared to a higher GI diet. 

The observation of this study is of great relevance to populations consuming white rice on a daily basis, and whose BMI is placing them at a high risk of developing diabetes. The literature reports that the consumption of white rice was associated with an increased risk of type 2 diabetes [32]. Substituting the consumption of white rice varieties with basmati rice or brown rice can aid them in attenuating their daily glucose levels and reducing their risk of developing diabetes in the future. 

Prior to the consumption of the mixed meals and usage of rtCGM, most participants preferred consuming the WRM. The results of this study are supported by findings from other studies [34]. The participants’ preference for mixed meals changed significantly after observing a steadier and lower increase in postprandial glucose level after consuming PBRM compared to the WRM, as the basmati rice meal was observed to be healthier based on its effect on the participants’ glucose levels. This was then translated into their daily food intake, which was recorded by the rtCGM. The peaks for the postprandial glucose levels were lower after the education using rtCGM took place. With reference to the follow-up, more than half of the participants included lower GI rice varieties in their diet as a result of observing their personalised postprandial response measured by the rtCGM in real-time. The outcome of this indicates that there is potential in utilising rtCGM among overweight and obese young adults as a learning tool, providing real-time information to unravel how different meals affect their glucose levels. This knowledge can assist them in selecting healthier food choices, such as lower GI foods, which can then be translated into their food habits. Although this study proves that rtCGM influenced the participants to select lower GI foods for a short duration, and participants claim to have made sustained improvements in their rice selection during follow-up, it needs to be proven in terms of real-life practice in their dietary intake by using validated methods. Future randomised controlled trials, which include a wider range of low GI index foods, larger sample size, and longer duration, need to be conducted to determine whether the use of rtCGM among this group of individuals will educate and motivate them to make more informed decisions on their meal intake, which can be construed as behavioural changes of their dietary pattern. 

The novelty of this study is that the GI of three varieties of commonly consumed rice were measured in a form of mixed meals replicating the way they are eaten on a daily basis in this region in a group of overweight and obese young adults instead of normal weight adults. This study also translates the feasibility of conducting randomised control trials using rtCGM among this group of individuals who are at a high risk of developing type 2 diabetes. Limited studies have observed the effect different GI meals have on the glycaemic response among overweight and obese young adults, specifically in the Asian region, as the literature suggests that GI may differ according to ethnicity [14,15]. The continuous 24 h glucose levels were measured while participants carried out their normal daily routines, allowing the results to be more reflective of real-life conditions. To our knowledge, this is the first study to determine the potential use of rtCGM system as an educating tool among overweight and obese young adults to assist and influence them in selecting lower GI foods to be practiced in their daily dietary intake. The real-time model of CGM allows the direct observation of the individual’s physiology in real-time. Individualised postprandial responses to a variety of foods can be easily recorded, which in turn can be used as a foundation to develop mixed meal menus for individuals to ensure that their blood glucose levels are under control while consuming a balanced diet. 

One of the limitations of this study is that the postprandial glucose level was solely measured by rtCGM and not by venous or capillary blood measurements. However, research utilising the same rtCGM model used in this study reported that the glucose levels measured by rtCGM, capillary blood, and venous blood testing were comparable, showing no significant difference in the results [17]. A single meal from each GI category was tested, limiting the generalisability of the outcome of this study. Future research should investigate more carbohydrate-based meals and observe the glycaemic responses of overweight and obese young adults towards these meals.

## 5. Conclusions

In conclusion, the GIs of three varieties of rice-based mixed meals were successfully determined by rtCGM. The lower GI basmati rice meal was shown to reduce the 24 h glucose level and GV among this population. This present study suggests that the rtCGM system can potentially be used as a new technique in measuring the GI of foods as it is convenient to use and is more sophisticated, as it produces more robust data continuously over 24 h and is less invasive. The rtCGM system can also potentially be used as an educating tool producing real-time information for overweight and obese individuals to select lower GI foods in their daily diet.

## Figures and Tables

**Table 1:** Nutrient composition and serving sizes of mixed meals.

Ingredient	Quantity /Amount (g)	Carbohydrate (g)	Protein (g)	Fats (g)	Fiber (g)	Rice-to-Water Ratio
**Rice**						
White rice	130	50	3.7	1.1	2.6	1:1
Brown rice	195	50	6.0	1.7	3.75	1:2
Parboiled basmati rice	165	50	4.3	0.9	1.7	1:1.5
**Grilled chicken**	100	-	30.5	3.2	-	n/a
**Salad**						
Lettuce	50	1.4	0.6	-	0.3	n/a
Tomato	25	0.9	0.4	-	0.1	n/a
Cucumber	25	0.8	0.1	-	0.2	n/a

Abbreviation: n/a, not applicable; -, does not contain or contains the specific nutrient at minimal amount.

**Table 2:** Characteristics of the study participants.

Characteristic	Mean ± SD
Sex Female, *n* (%) Male, *n* (%)	5 (35.7) 9 (64.3)
Age (years)	22.8 ± 4.6
Height (cm)	165.0 ± 9.9
Weight (kg)	89.8 ± 18.9
Body mass index (kg/m^2^)	32.9 ± 5.8
Fat (%)	35.9 ± 7.9
Fat mass (kg)	31.9 ± 8.4
Fat free mass (kg)	57.8 ± 15.1
Muscle mass (kg)	54.8 ± 14.5
Total body water (%)	46.6 ± 3.9
Bone mass (kg)	3.1 ± 0.7
Visceral fat rating	12.9 ± 3.9
Fasting glucose level (mmol/L)	4.9 ± 0.4
^¶^ Estimated A1c (%)	4.7 ± 0.3

Values are mean (SD), unless otherwise stated. **^¶^** Value estimated using rtCGM.

**Table 3:** IAUC and GI of meals of different varieties of rice measured using the real-time continuous glucose monitoring system (rtCGM).

Mixed Meals	IAUC, mmol-min/L (Mean ± SD)	Measured GI (Mean ± SD)	* Predicted GI	^¶^ GI Categories
Reference drink	208.08 ±13.66	100		-
WRM	173.19 ± 9.86 ^a^	83.03 ± 2.19 ^a^	77	High
BRM	120.93 ± 5.20 ^b^	56.44 ± 2.34 ^b^	52	Medium
PBRM	97.77 ± 6.02 ^c^	45.35 ± 2.06 ^c^	48	Low

Data were analysed by one-way ANOVA and post hoc Tukey’s test. Different superscript letters (a–c) indicate the values that are statistically significant at each time point at *p* ≤ 0.05. * Predicted meal GI is calculated as described by Dodd and colleagues [13] using GI values obtained from the University of Sydney Glycaemic Index Research and GI News [28]. ^¶^ GI categories: low ≤ 55, medium: 56–69, high ≥ 70.

**Table 4:** The 24 h mean glucose level, 24 h glucose target ranges and glycaemic variability of reference drink and mixed meals.

Reference Drink or Mixed Meals	Glycaemic Variability, % CV (Mean ± SD)	24 h Mean Glucose Level, mmol/L (Mean ± SD)	24 h Glucose Target Range (Mean% ± SD)		
			Below Target	In Target	Above Target
Reference drink	20.8 ± 5.2 ^a^	5.37 ± 0.54 ^b^	10.2 ± 13.8 ^b^	86.9 ± 14.1 ^ab^	2.9 ± 4.3 ^a^
WRM	18.9 ± 4.6 ^a^	5.29 ± 0.62 ^b^	11.3 ± 18.2 ^b^	85.6 ± 17.3 ^a^	3.1 ± 4.1 ^a^
BRM	19.8 ± 3.8 ^a^	5.03 ± 0.45 ^ab^	8.6 ± 13.5 ^ab^	88.5 ± 12.2 ^ab^	2.9 ± 3.2 ^a^
PBRM	17.2 ± 4.7 ^a^	4.87 ± 0.66 ^a^	7.6 ± 9.2 ^a^	90.1 ± 9.6 ^b^	2.3 ± 3 ^a^

Different superscript letters (a, b) indicate that values are statistically significant at *p* ≤ 0.05 within the same columns. WRM: white rice meal; BRM: brown rice meal; and PBRM: parboiled basmati rice meal. The 24 h glucose target range; below target level (<3.9 mmol/L); in target level (3.9–7.8 mmol/L); and above the target level (>7.8 mmol/L) [29].

**Table 5:** Mixed meal preference before and after observing the trendline of the postprandial glucose levels indicated by rtCGM.

Mixed Meals	Mixed Meal Preference		*p*-Value
	Before *n* = 14 (%)	After *n* = 14 (%)	
WRM	9 (64.3)	0 (0)	
BRM	2 (14.3)	4 (28.6)	0.012 *
PBRM	3 (21.4)	10 (71.4)	

* Significant difference at *p* < 0.05. WRM: white rice meal; BRM: brown rice meal; and PBRM: parboiled basmati rice meal.

## Data Availability

The datasets used and/or analysed during the current study are available from the corresponding author on reasonable request.
